# Identification of an Imprinted Gene Cluster in the X-Inactivation Center

**DOI:** 10.1371/journal.pone.0071222

**Published:** 2013-08-06

**Authors:** Shin Kobayashi, Yasushi Totoki, Miki Soma, Kazuya Matsumoto, Yoshitaka Fujihara, Atsushi Toyoda, Yoshiyuki Sakaki, Masaru Okabe, Fumitoshi Ishino

**Affiliations:** 1 Japan Science and Technology Agency, Precursory Research for Embryonic Science and Technology, Kawaguchi, Saitama, Japan; 2 Department of Epigenetics, Medical Research Institute, Tokyo Medical and Dental University, Bunkyo-ku, Tokyo, Japan; 3 Division of Cancer Genomics, National Cancer Center Research Institute, Chuo-ku, Tokyo, Japan; 4 Research Institute for Microbial Diseases, Osaka University, Suita, Osaka, Japan; 5 Comparative Genomics Laboratory, Center for Information Biology, National Institute of Genetics, Mishima, Shizuoka, Japan; 6 Toyohashi University of Technology, Toyohashi, Aichi, Japan; Wellcome Trust Centre for Stem Cell Research, United Kingdom

## Abstract

Mammalian development is strongly influenced by the epigenetic phenomenon called genomic imprinting, in which either the paternal or the maternal allele of imprinted genes is expressed. Paternally expressed *Xist*, an imprinted gene, has been considered as a single *cis*-acting factor to inactivate the paternally inherited X chromosome (Xp) in preimplantation mouse embryos. This means that X-chromosome inactivation also entails gene imprinting at a very early developmental stage. However, the precise mechanism of imprinted X-chromosome inactivation remains unknown and there is little information about imprinted genes on X chromosomes. In this study, we examined whether there are other imprinted genes than *Xist* expressed from the inactive paternal X chromosome and expressed in female embryos at the preimplantation stage. We focused on small RNAs and compared their expression patterns between sexes by tagging the female X chromosome with green fluorescent protein. As a result, we identified two micro (mi)RNAs–miR-374-5p and miR-421-3p–mapped adjacent to *Xist* that were predominantly expressed in female blastocysts. Allelic expression analysis revealed that these miRNAs were indeed imprinted and expressed from the Xp. Further analysis of the imprinting status of adjacent locus led to the discovery of a large cluster of imprinted genes expressed from the Xp: *Jpx*, *Ftx* and *Zcchc13*. To our knowledge, this is the first identified cluster of imprinted genes in the *cis*-acting regulatory region termed the X-inactivation center. This finding may help in understanding the molecular mechanisms regulating imprinted X-chromosome inactivation during early mammalian development.

## Introduction

In mammalian development, X-chromosome inactivation (XCI) as well as genomic imprinting is a well-known epigenetic regulatory mechanism [1], [2]. In female mammals, XCI maintains the level of gene expression from both X chromosomes to be the same as in male mammals. Without XCI, female embryos die, indicating the importance of this epigenetic regulation in female mammalian development [3]. During mouse preimplantation development, the paternally inherited X chromosome (Xp) derived from the fertilizing spermatozoon begins to be inactivated selectively by imprinted XCI. This imprinted inactivation is completely established in the extraembryonic tissues after implantation [4]–[7].

To date, *Xist*, an imprinted gene expressed from the inactive Xp, has been thought to be a single *cis*-acting regulator of imprinted XCI. However, the mechanism has not been elucidated fully at a molecular level and there is little information about imprinted genes expressed from the inactivated Xp. Therefore, we examined whether there are imprinted genes, other than *Xist*, expressed from the Xp and only detectable in female preimplantation embryos. If such imprinted genes exist, they are likely candidate genes involved in the process of imprinted XCI.

We have compared the expression patterns of coding genes in wild-type male and female blastocysts using DNA microarrays that cover all known genes. From the comparison, *Rhox5* and *Fthl17* were identified as imprinted genes predominantly expressed in female embryos and expressed from the inactive Xp [8], [9]. These two genes are the only candidates other than *Xist*. However, targeted disruption of *Rhox5* as well as interfering RNA (RNAi) knockdown of the *Fthl17* gene did not affect the birth of female mice, suggesting that these candidate genes are dispensable for the mechanism regulating imprinted XCI [10].

In addition to such coding genes, several studies have indicated the involvement of noncoding RNAs (ncRNA), including small RNAs, in regulating gene expression [11]–[14]. Furthermore, micro (mi)RNAs and small nucleolar (sno)RNAs are imprinted and proposed to be involved in mammalian development [15]. These findings led us to speculate on the possible involvement of imprinted ncRNAs in the regulation of XCI. Using pyrosequencing, we compared expression patterns of small RNAs between male and female preimplantation embryos. Here, we report that two X-linked miRNAs are imprinted and form a large cluster of imprinted genes expressed from the Xp at the preimplantation stage. As far as we know, this is the first identified imprinted gene cluster located within the X-inactivation center that is known to regulate XCI.

## Materials and Methods

### Animals

The handling and surgical manipulation of all experimental animals were carried out according to the guidelines of the Committee on the Use of Live Animals in Teaching and Research of Tokyo Medical and Dental University, and animal protocols were reviewed and approved by the animal welfare committee of the Tokyo Medical and Dental University (Permit Number: 0130228A). All efforts were made to minimize suffering. The B6C3F1 TgN (act EGFP) Osb CX-38 (G38) transgenic mouse strain expressing the transgene for enhanced green fluorescent protein (EGFP) described elsewhere [8], [9], [16] was used to distinguish between male and female embryos.

### Blastocyst Collection for Sequencing Small RNAs

Eight-week-old B6C3F1 female mice were superovulated with intraperitoneal injections of 5 IU pregnant mare serum gonadotropin followed by 5 IU of human chorionic gonadotropin (hCG) 48 h later. These superovulated female mice were mated with X^GFP^Y male mice. Embryos at the 4-cell stage were collected from the oviducts 55 h after the hCG injection, placed in potassium simplex optimization medium and incubated in a humidified atmosphere of 5% CO_2_ in air at 37°C for an additional 38 h. After collecting early to mid-stage blastocysts, male (EGFP-negative) and female (EGFP-positive) embryos were separated using a fluorescence microscope (M165FC; Leica Microsystems GmbH, Wetzlar, Germany).

### Small RNA Library Construction and Sequencing

Small RNA libraries were constructed as described [17]. Briefly, small RNAs (18–26 mer) were isolated from male and female blastocysts: 2000 each. The libraries of male and female blastocysts were constructed using a small RNA cloning kit (TaKaRa Bio Inc., Ohtsu, Japan) and the sequences of small RNAs were determined using a 454 Life Sciences pyrosequencer (Branford, CT). The small RNAs were mapped to the genome using the NCBI BLASTN program (http://blast.ncbi.nlm.nih.gov). Perfect match sequences were selected for further analysis. To annotate small RNAs, we used the following databases: miRNA_mature and miRNA_hairpin from miRBasev16; rRNA, snRNA, snoRNA, lincRNA, miscRNA, Mt_rRNA and Mt_tRNA, from Ensembl (release 6). Of the 397,726 and 473,043 mapped reads from the female and male libraries, only around 10% have been confirmed as noncoding RNAs, the remaining ∼90% are classified “only genomic” (Table 1). Therefore, further annotation was carried out using these “only genomic” sequences against RepeatMasker (mm9 - Jul 2007 - RepeatMasker open-3.2.8 - Repeat Library 20090604) and the mouse tRNA database (tRNAscan-SE Analysis of *Mus musculus* (mm9 July 2007)). As a result, ∼70% of the sequences were matched to repeat or tRNA sequences, suggesting that some of the small RNAs were degradation products derived from these sequences (Table S1). RNA abundance was measured using reads per kilobase per million reads (RPKM) [18]. The expression data of these annotated small RNAs are supplied as Table S2. The small RNA sequence data derived from male and female blastocysts have been deposited in the DNA Data Bank of Japan (http://www.ddbj.nig.ac.jp) under the accession number DRA001023.

**Table 1:** Classification of annotated small RNAs in male and female blastocysts.

	Female	Male
No. reads	433,905	517,507
Genome mapped*	397,726	473,043
miRNA_mature	31,488	31,608
Mt_tRNA	3,609	4,247
Mt_rRNA	1,637	2,339
snRNA	1,142	1,510
rRNA	1,026	1,180
snoRNA	1,055	1,100
misc_RNA	381	486
lincRNA	402	479
miRNA_hairpin	130	167
(Total ncRNA)	40,876	43,121
Only genomic**	363,003	437,224

* The number of genes mapped is not always equal to the “Total ncRNA” plus “Only genomic” counts because of multiple annotations for single read sequences.

Abbreviations: miRNA_mature, micro RNA mature form; Mt_tRNA, mitochondrial transfer RNA; Mt_rRNA, mitochondrial ribosomal RNA; snRNA, small nuclear RNA; rRNA, ribosomal RNA; snoRNA, small nucleolar RNA; miscRNA, miscellaneous RNA; lincRNA, large intergenic noncoding RNA; miRNA_hairpin: micro RNA stem-loop sequence obtained from miRBase.

** The sequences classified as “Only genomic” were further annotated against RepeatMasker and the tRNA database. The results are shown in Table S1.

### Quantitative Reverse Transcription Polymerase Chain Reaction (qRT–PCR) Analysis of Candidate Genes and Small RNAs for Sexually Differential Expression

The total RNA contents of male and female blastocysts were extracted using TRIzol (Invitrogen, Carlsbad, CA) and any contaminating DNA was digested using RNase-free DNase (Nippon Gene, Tokyo, Japan). In the case of qRT–PCR for mRNAs, first-strand cDNA was synthesized using reverse transcriptase (SuperScript III; Invitrogen) and random primers according to the manufacturer’s instructions. Expression levels are reported as the ratios of the number of cDNA copies for each gene to that of cDNA copies for the housekeeping gene *β-actin*. Primers used for qRT–PCR are summarized in Table S2. For miRNA expression analysis, total RNA was reverse transcribed using the TaqMan MicroRNA Reverse Transcription Kit (Applied Biosystems, Foster City, CA). The TaqMan MicroRNA assays used (with identification numbers in brackets), were as follows: mmu-miR-374-5p (001319); mmu-miR-421-3p (002700); mmu-miR-107-3p (000443); mmu-miR-103-3p (000439); mmu-miR-30c-5p (000419); mmu-miR-7a-5p (000268); mmu-miR-19b-3p (000396); mmu-miR-467c-3p (464331); mmu-miR-295 (000189); snoRNA202 (001232). Mature miRNAs were amplified with TaqMan Universal PCR Master Mix II (Applied Biosystems) using a LightCycler 480 instrument (Roche Diagnostics, Mannheim, Germany). The qRT–PCR reactions were performed using samples collected from three independent RNA preparations.

### Verification of Imprinting

Two reciprocal sets of hybrid blastocysts, (JF1× C57BL/6) F1 and (C57BL/6× (JF1× C57BL/6)) F1, were produced by cross-mating appropriately. All samples were recovered from the uteri to avoid the effects of embryo culture. Genomic DNA and RNA were extracted from individual blastocysts. Male and female blastocysts were pooled separately after sex determination by PCR as described [8]. In each experiment, at least 60 blastocysts were pooled by sex. One third of the resulting cDNA samples derived from pooled RNAs were amplified by PCR using the primers listed in Table S2.

### *In situ* Hybridization (ISH)

Whole-mount ISH was performed as described [19]. Briefly, a full-length Ftx variant 2 cDNA clone (RIKEN FANTOM clone ID: 9530061G23) was used for preparation of digoxigenin-labeled sense and antisense RNA probes using the DIG RNA Labeling Mix (Roche, Basel, Switzerland). Specific signals were detected using an alkaline phosphatase-conjugated anti-digoxigenin antibody (Roche).

### Statistics

Two-tailed Student’s *t*-tests were used to analyze the significance of any differences and *P*<0.01 was considered significant. All quantitative data are presented as the mean ± standard deviation of the mean.

## Results

### Identification of Small RNAs Predominantly Expressed in Female Embryos

**Figure 1:**
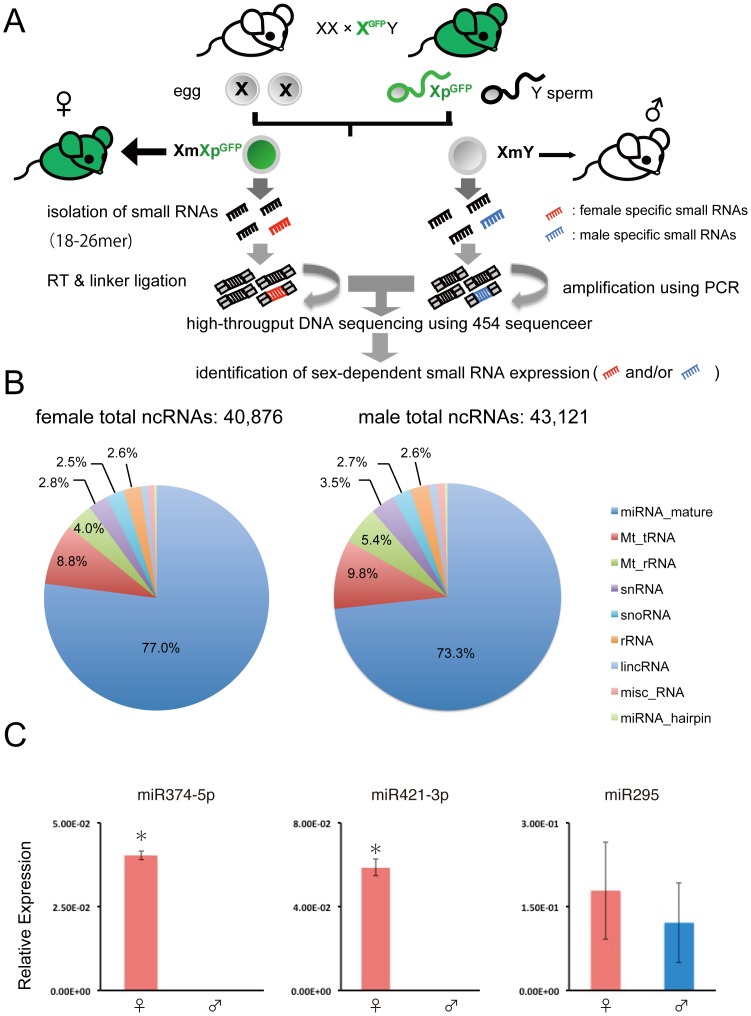
Identification of small RNAs predominantly expressed in female blastocysts. (A) Scheme of the X^GFP^ sexing system and construction of small RNA libraries. Transgenic male mice (X^GFP^Y) were mated with wild-type female mice (XX). Only the female embryos (XX^GFP^) fluoresce green because of the paternally inherited X^GFP^. Small RNAs obtained from male and female blastocysts were sequenced and expression levels were compared between sexes. (B) Annotation of the read sequences from male and female blastocyst libraries. The distribution of the RNA classes is given as a percentage of the total annotated ncRNAs. (C) Expression levels of miR-374-5p, miR-421-3p and miR-295 miRNA in male and female blastocysts. The expression of three miRNAs was measured by qRT–PCR and normalized to that of snoRNA202 examined as a control. The data indicate that the miR-374-5p and miR-421-3p were predominantly expressed in female blastocysts (*P<0.01). Results are expressed as the mean ± SD (*n* = 3).

To screen small RNAs predominantly expressed in female embryos, we used a transgenic mouse line in which the X chromosome is tagged with the transgene for EGFP [8], [9], [20]. The sexing methodology is shown in Fig. 1A. The transgenic males (X^GFP^Y) were mated with wild-type females (XX). Because of the technical difficulty of separating early male and female embryos using X-GFP mice, we chose blastocysts in which we could separate the sexes very easily and accurately.

**Figure 2:**
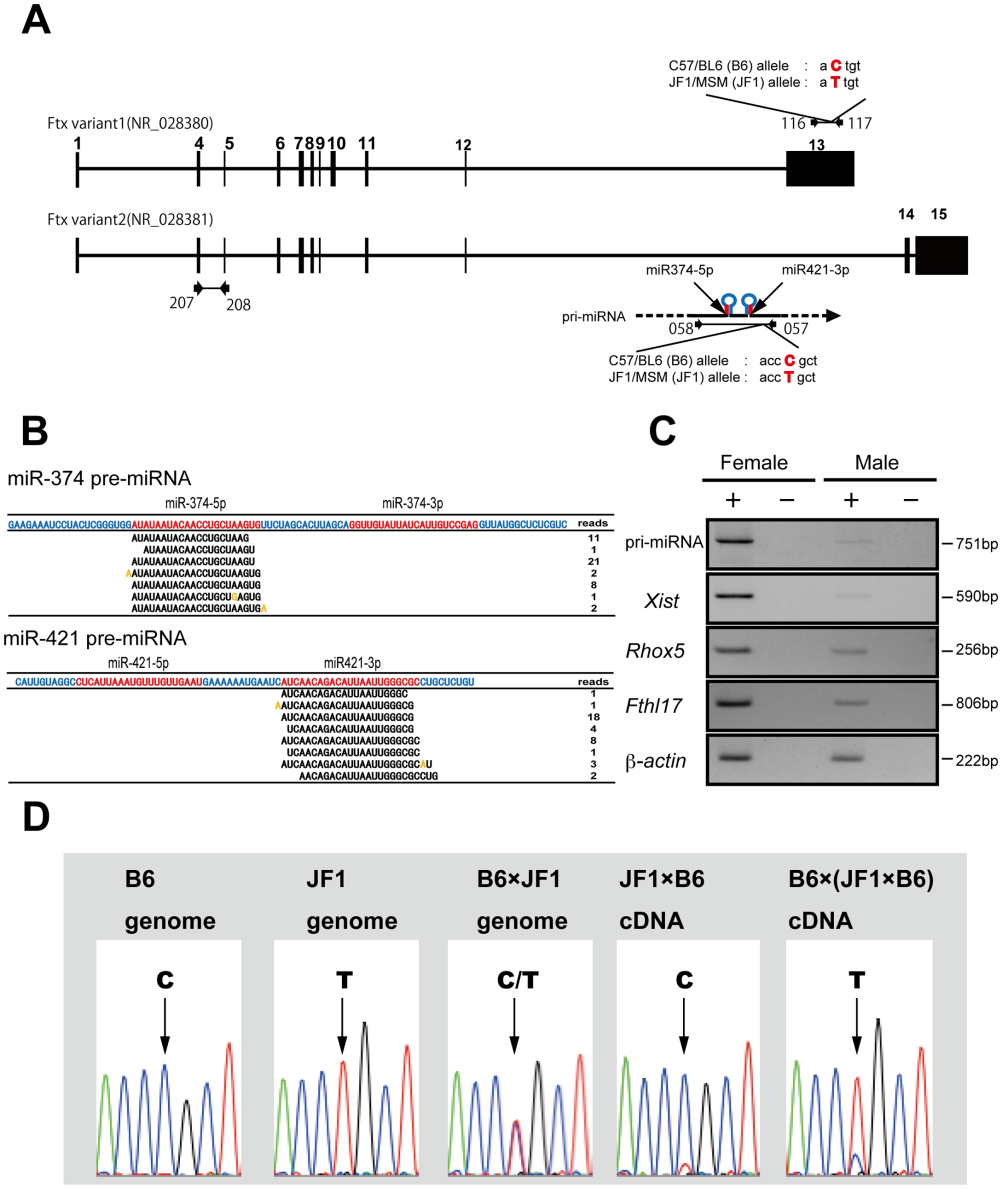
Verification of imprinting of two miRNAs predominantly expressed in female embryos. (A) Schematic representation of two mature miRNAs, miR-374-5p and miR-421-3p and their preceding transcript pri-miRNA. Both miRNAs were clustered and located in the intron of the *Ftx* (B230206F22Rik) gene on the X chromosome. Both are thought to be transcribed as pri-miRNAs and then processed to form mature miRNAs. The location of the primers used to assess the pri-miRNA and *Ftx* expression levels are indicated: qRT–PCR primers 207 and 208 were used for *Ftx*; primers used for allelic discrimination were 057 and 058 for pri-miRNA, 116 and 117 for *Ftx*. The exons for *Ftx* are numbered according to a previous report [22]. DNA polymorphisms used for allelic discrimination are indicated with red characters. PCR primers correspond to the list in Table S3 (B) Production of mature miRNAs from pre-miRNAs in female blastocysts. Although two mature forms of miRNA, miR-374-5p/miR-374-3p and miR-421-5p/miR-421-3p are registered in miRBase, only miR-374-5p and miR-421-3p were detected at the blastocyst stage. Orange coloring shows mismatch sequences compared with the reference sequences, suggesting miRNA editing or misreading by the 454 sequencer. (C) Expression of pri-miRNA in wild-type male and female blastocysts obtained from the uterus. Female-predominant expression was observed as well as previously reported X-linked imprinted genes such as *Xist and Rhox5*. (D) Verification of imprinting of the pri-miRNA for miR-374-5p and miR-421-3p in preimplantation mouse embryos. Pri-miRNA was expressed from the paternal B6 allele in JF1× C57BL/6 crosses and from the paternal JF1 allele in the reciprocal B6× (JF1× B6) cross. The arrows indicate the polymorphism sites that were used in this assay.

First, small RNAs were isolated individually from male and female blastocysts and compared using 454 pyrosequencing followed by sequence annotation. We obtained 397,726 male and 473,043 female small RNA sequences, which were matched with the mouse genome completely. Table 1 shows the annotated small RNA sequences using miRbase (v. 16) and the Ensembl database (release 61). There was no apparent difference in the total distribution of annotated small RNAs between the two sexes (Fig. 1B; Table 1). Therefore, we next compared the expression patterns of individual small RNA sequences and discovered a number of differences between the sexes (Table 2, 3). To confirm the results of pyrosequencing, we carried out qRT–PCR of small RNAs showing more than 10-fold (1000%) changes in sequence reads. We successfully identified two miRNAs, miR-374-5p (Accession number: MI0004125 in miRBase) and miR-421-3p (MI0005496) that were expressed predominantly in female blastocysts(Fig. 1C). There was no significant difference of expressions of other miRNAs between sexes (Fig. S1). We also showed quantification of a microRNA (miR295: MI0000393) not submitted to imprint as control. There was no significant difference of expression between sexes (Fig. 1C). Interestingly, the two mature miRNAs (miR-374-5p and miR-421-3p) we identified were mapped close together on the X chromosome and appeared to be derived from a single primary (pri) miRNA (Fig. 2A). This was predicted to produce two stem–loop structured precursor (pre)-miRNAs. According to miRBase, each of these two pre-miRNAs produces two mature miRNAs, miR-374-5p/miR-374-3p and miR-421-5p/miR-421-3p (Fig. 2B). However, in our study each of the two pre-miRNAs produced a single mature miRNA, miR-374-5p and miR-421-3p, respectively, and neither miR-374-3p nor miR-421-5p was detected at the blastocyst stage.

**Table 2:** List of differentially expressed small RNAs between male and female blastocysts (1).

Upregulated small RNAs in female blastocysts.					
RNA_ID	RNA_TYPE	Female_READS*	Male_READS*	M/F_READS	Map position (chromosome number)
mmu-miR-374-5p	miRNA_mature	46.00	–	0.000	X
mmu-miR-421-3p	miRNA_mature	38.00	–	0.000	X
ENSMUST00000143469/Gm13448	lincRNA	13.00	–	0.000	2
**RNA_ID**	**RNA_TYPE**	**Female_READS** **	**Male_READS** **	**M/F_READS**	**Map position (chromosome number)**
mmu-miR-107	miRNA_mature	32.94	0.01	0.000	19
mmu-miR-103	miRNA_mature	71.53	0.04	0.001	11
mmu-miR-30c	miRNA_mature	181.67	0.13	0.001	4
mmu-miR-7a	miRNA_mature	29.00	0.03	0.001	13
mmu-miR-19b	miRNA_mature	53.00	0.11	0.002	X
ENSMUST00000093731	snoRNA	95.99	0.81	0.008	11
ENSMUST00000082466	snoRNA	48.02	0.60	0.013	19
ENSMUST00000082873	snoRNA	25.61	0.61	0.024	11

* Sequence reads of small RNAs that showed 0 reads in male embryos and more than 10 reads in female embryos.

** Small RNAs showing more than a 10-fold (1000%) change in sequence reads are listed. Small RNAs showing fewer than 20 reads have been excluded from this list.

**Table 3:** List of differentially expressed small RNAs between male and female blastocysts (2).

Upregulated small RNAs in male blastocysts.					
RNA_ID	RNA_TYPE	Female_READS†	Male_READS†	M/F_READS	Map position (chromosome number)
mmu-miR-467c*	miRNA_mature	0.02	23.98	1372.867	2
ENSMUST00000083244	snRNA	0.71	69.49	98.219	13
ENSMUST00000083111	snRNA	2.72	99.33	36.580	3
ENSMUST00000082989	snRNA	7.00	137.46	19.625	4

† Small RNAs showing more than a 10-fold (1000%) change in sequence reads are listed. Small RNAs showing fewer than 20 reads have been excluded from this list.

### Verification of Imprinting of the Female X Chromosome-expressed miRNA

We next examined whether miR-374-5p and miR-421-3p were imprinted, as is the case for *Xist* [21]. First, to distinguish paternal expression from maternal expression, we searched for DNA polymorphisms between *Mus mus domesticus* (C57BL/6: B6) and the Japanese wild mouse *M. m. molossinus* (JF1/MSM). Mature forms of miRNAs are only 22- and 23-nucleotides (nt) long, making it difficult to find polymorphisms within their sequences. We utilized a polymorphism identified in the pri-miRNA and examined the active alleles in F1 blastocysts. Then, we confirmed that the pri-miRNA in F1 blastocysts was also predominantly expressed in female embryos as well as the mature two miRNAs (Fig. 2C). Furthermore, allelic expression analysis revealed that the pri-miRNA was expressed from the Xp in F1 blastocysts (JF1× B6) derived from mating between JF1/MSM females and C57BL/6 males and F1 blastocysts (B6× (JF1× B6)) derived from the reciprocal cross of C57BL/6 females and F1 males (JF1/MSM × C57/BL6) (Fig. 2D). These data confirmed that miR-374-5p and miR-421-3p are both imprinted and expressed from the Xp chromosome.

### Identification of a Novel Cluster of Imprinted Genes on the X Chromosome

**Figure 3:**
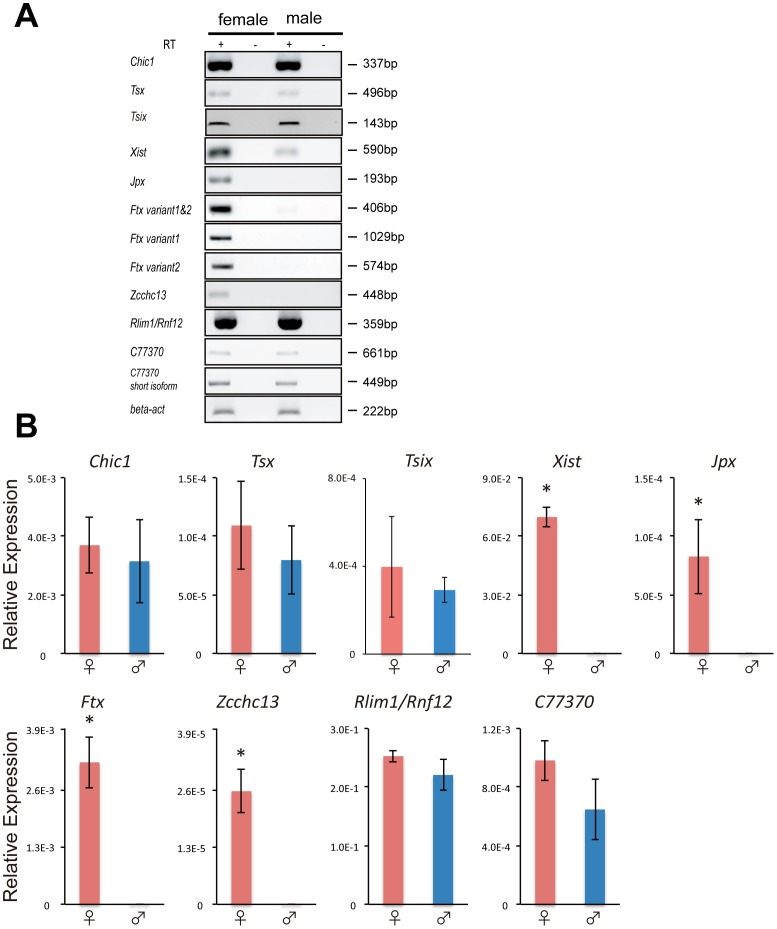
Searching for a cluster of imprinted genes on the X chromosome. (A) qRT–PCR of X-linked genes in male and female blastocysts. It should be noted that very weak expression levels of *Xist, Jpx, Ftx, Zcchc13* were detected in male blastocysts when additional PCR cycles were carried out. (B) The expression levels of *Chic*, *Tsx*, *Xite, Tsix, Xist*, *Jpx*, *Ftx*, *Zcchc13*, *Rlim1/Rnf12*, *Slc16a2*, *C77370* were quantified by qRT–PCR in female and male mouse blastocysts. The ratios of the number of cDNA copies for each gene to that of cDNA copies for a housekeeping gene, *β-actin*, are indicated as expression levels. Expressions of *Xite* and *Scl16a2* were not detected at the blastocyst stage (data not shown). Results are expressed as the mean ± SD (*n* = 3). The statistical significance of differences between sample means was determined by Student’s *t*-test. *P<0.01.

**Figure 4:**
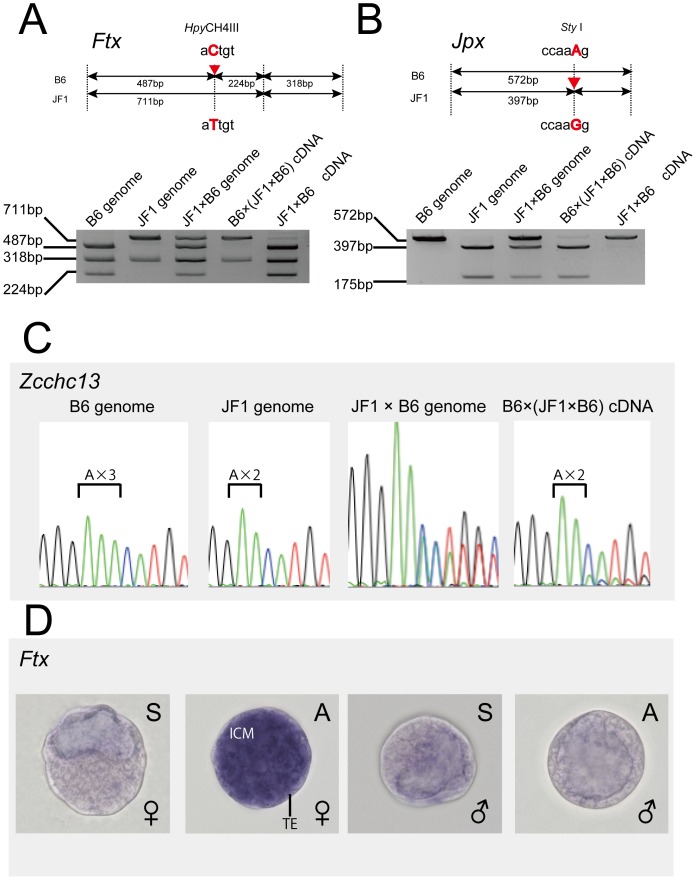
Allelic expression analysis of female-expressed genes at the blastocyst stage. (A) Verification of imprinting in the *Ftx* gene. Upper: schematic representation of the position of the DNA polymorphism in the *Ftx* gene. The single nucleotide polymorphism detected in C57BL/6: B6 (aCtgt) and JF1/Ms: JF1 (aTtgt) is shown. The Hpy CH4III site in the B6 allele was changed in the JF1 allele. These alleles could be distinguished by digestion with *Hpy* CH4III. Lower: the imprinted expression of *Ftx* from the Xp chromosome was determined by qRT–PCR and restriction fragment length polymorphism (RFLP) analysis with inter-subspecific hybrid mouse F1 progenies. (B) Verification of *Jpx* imprinting. Upper: schematic representation of the position of the DNA polymorphism in the *Jpx* gene. The *Sty* I site in the B6 allele (ccaaAg) was changed in the JF1 allele (to ccaaGg). Lower: imprinted expression of *Jpx* from the Xp chromosome was determined by qRT–PCR and RFLP analysis with inter-subspecific hybrid mouse F1 progenies. (C) Verification of the *Zcchc13* imprinting. The single nucleotide insertion polymorphism detected in C57BL/6; B6 (triple A) and JF1/Ms; and JF1 (double A) is shown. The imprinted expression of *Zcchc13* from the Xp chromosome was determined by sequencing analysis with inter-subspecific hybrid mice F1 progenies. (D) Whole-mount *in situ* hybridization of the *Ftx* gene in female and male blastocysts. ‘S’ indicates the sense strand probe and ‘A’ indicates the antisense strand probe. Abbreviation: ICM, Inner cell mass; TE, trophectoderm.

Given that most autosomal imprinted genes exist as clusters, we considered the possibility that these imprinted miRNA might cluster with other imprinted genes on the X chromosome. The two imprinted miRNAs were indeed mapped adjacent to the imprinted *Xist* gene, strengthening the possibility that they might form a cluster of imprinted genes. Therefore, we analyzed the expression patterns of genes neighboring these miRNAs. From the neighborhood region of approximately 1 Mb of miRNA (UCSC mm9), the expression level of 11 genes–*Chic1*, *Tsx*, *Xite, Tsix, Xist*, *Jpx/Enox*, *Ftx/B230206F22Rik*, *Zcchc13*, *Slc16a2*, *Rlim/Rnf12* and *C77370*–were compared between male and female blastocysts. In addition to *Xist*, three genes, *Ftx*, *Jpx* and *Zcchc13*, were expressed predominantly in female blastocysts (Fig. 3A, B). We next examined the allelic expression of these genes to verify whether they were imprinted. *Ftx* is a polyadenylated ncRNA with two different spliced variants (NR_028380 and NR_028381) [22]. Both variants were predominantly expressed in female embryos (Fig. 3A). Using the DNA polymorphism found in the last exon of *Ftx* variant 1 (NR_028380), we verified that the *Ftx* gene was expressed predominantly when it was derived from the father (Fig. 4A). Similarly, the allelic expression of *Jpx* and *Zcchc13* clearly showed that these two genes are also imprinted and expressed from the Xp chromosome (Fig. 4B, C). Taken together, these female-expressed imprinted genes on the X chromosome form a cluster adjacent to the miRNAs, miR-374-5p and miR-412-3p.

To investigate the association between the expression patterns of newly identified imprinted genes and X-chromosome inactivation further, whole-mount ISH was carried out on blastocysts. Unfortunately, the expression levels of *Jpx* and *Zcchc13* were too low to be detected by ISH. In contrast, *Ftx*, the most highly expressed gene among the identified imprinted genes, gave strong signals in female embryos in the trophectoderm where the Xp chromosome is inactivated and the ICM where the Xp chromosome is reactivated (Fig. 4D), whereas a signal was barely detectable in male blastocysts. Notably, *Ftx* was imprinted and expressed from the Xp chromosome, although this is selectively inactivated in the trophectoderm.

## Discussion

### A Cluster of Imprinted Genes on the X Chromosome is Expressed in Female Preimplantation Mouse Embryos

**Figure 5:**
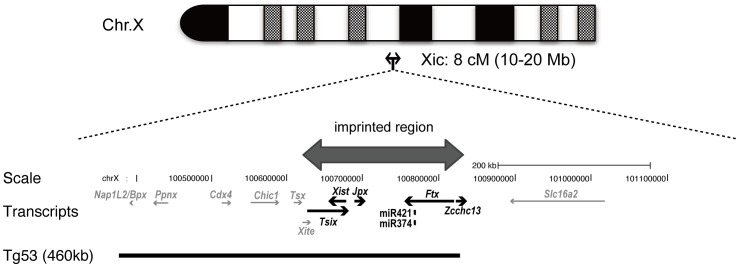
Identification of a cluster of imprinted genes in the X-inactivation center (Xic). A newly identified cluster of imprinted genes on the X chromosome is shown in the mouse genome (UCSC mm9; http://genome.ucsc.edu/). This cluster is located within the Xic. The arrows show the direction of each transcription unit. *Ftx* and Z*cchc13* are transcribed in opposite directions from the same putative bidirectional promoter. Imprinted genes are shown by the thick black arrows; *Xist, Jpx, Ftx, Zcchc13*, miR-374-5p and miR-421-3p were paternally expressed, while *Tsix* was reported previously to be maternally expressed [28], [29]. The thick black bar indicates the position of the Tg53 (460 kb) transgene reported in [35].

Here we found imprinted genes existing as a cluster and expressed from the Xp chromosome in preimplantation embryos. This suggests that these imprinted genes are additional candidates involved in imprinted XCI. Despite the early discovery of various imprinting effects observed including XCI [4], [5], [7], [23]–[26], there have been relatively few reports about imprinted genes on X chromosomes [27]. *Xist* was the first identified X-linked imprinted gene and is believed to play major roles in imprinted XCI. Adjacent to *Xist*, here we identified a number of imprinted genes including miR-374-5p and miR-421-3p and the long ncRNAs *Jpx* and *Ftx*, together with coding RNA *Zcchc13* as a CCHC-type Zinc finger protein. Other than these imprinted genes, *Tsix* was previously reported to be imprinted and expressed from the maternal X chromosome [28], [29]. Our findings mean that this imprinted gene cluster reaches ∼200 kbp in length and we have named it the *Tsix–Zcchc13* imprinted gene cluster (Fig. 5).

### Imprinting Mark of the *Tsix-Zcchc13* Imprinted Region

Allele-specific expression of imprinted genes requires an epigenetic mark to distinguish paternal from maternal chromosomes. Each cluster of imprinted genes is thought to be controlled by an imprinting control region (ICR) that is marked by DNA methylation during either oogenesis or spermatogenesis. Concerning the *Tsix–Zcchc13* imprinted region, it has not been known how the paternal expression of *Xist* is controlled. However, our results indicate that this might be considered as a problem of how the entire *Tsix–Zcchc13* imprinted region is controlled rather than a problem of how the single *Xist* gene is controlled. Genome-wide DNA methylation profiles from gametes have revealed that six CpG islands located within the *Tsix–Zcchc13* region show hypomethylated patterns and have no characteristic of differentially methylated regions that work as ICRs in other autosomal imprinted regions [30], [31]. Given that the paternal expression of *Xist* does not require DNA methylation [32], these data suggest that the imprinting of this newly identified X-linked region is established by DNA methylation-independent mechanisms. Identification of the imprinted *Tsix–Zcchc13* region should help us narrow down the genomic area to search for epigenetic marks distinct from DNA methylation.

Concerning the epigenetic state of this imprinted region, *Ftx* and *Jpx* have been reported to partially escape random XCI in differentiated ES cells [22], [33] and also to evade imprinted XCI in trophoblastic stem cells [34]. This suggests that the epigenetic state of this imprinted gene cluster differs from other inactivated X chromosome regions.

### Roles of the *Tsix–Zcchc13* Imprinted Gene Cluster in Imprinted XCI

Regarding the function of the identified imprinted gene cluster, Okamoto *et al*. indicated a possible involvement of this cluster in the regulation of imprinted XCI. A 460 kb single-copy transgene (*Tg53*) containing a part of the *Tsix–Zcchc13* cluster was reported to induce imprinted *cis*-inactivation in autosomes at the preimplantation embryo stage when it was inherited paternally [35]. Thus, this 460 kb transgene contains sufficient factors to trigger imprinted inactivation of the paternal chromosome. The 460 kb transgene was reported to encompass the genomic region from *Nap1l2/Bpx to Ftx*, but not *Zcchc13* (Fig. 5). Our research provides evidence that, in addition to the *Xist* gene, this transgene possesses two imprinted genes and two miRNAs: *Jpx*, *Ftx*, miR-374-5p and miR-421-3p that are expressed from the Xp chromosome. Thus, this transgenic analysis supported the idea that these newly identified imprinted genes (*Jpx and Ftx* and two miRNAs, miR-374-5p and miR-421-3p) function as *cis*-acting factor(s) and are involved in the mechanisms of imprinted paternal XCI at the preimplantation embryo stage.

### Common Machinery for Random and Imprinted XCI

XCI can be separated into two consecutive processes, initial ‘imprinted XCI’ followed by ‘random XCI’. Random XCI is controlled by a *cis*-acting X-inactivation center termed Xic. [36], [37]. Inside Xic, we found a large cluster of imprinted genes: the *Tsix–Zcchc13* cluster (Fig. 5). Among them, *Jpx* and *Ftx* were reported to upregulate the expression of *Xist* in random XCI mechanisms [22], [33]. Given the functions of these ncRNAs as positive regulators, they can also function to activate the expression of *Xist* in imprinted XCI at the preimplantation stage. Gene targeting and the generation of transgenic mice carrying these imprinted ncRNAs and miRNAs will help answer whether and how they are involved in imprinted XCI. Our finding not only provides much information about X-linked imprinted genes, but also helps in clarifying the molecular mechanisms of imprinted XCI. Functional analysis of these imprinted genes is currently under way using gene targeting technology.

## Supporting Information

Figure S1 **Validation of the expression of micro RNAs using qRT–PCR.** The qRT–PCR analysis of differentially expressed microRNA candidates mmu-miR-7a, mmu-miR-19b, mmu-miR-30c, mmu-miR-103, mmu-miR-107 and mmu-miR-467 that are presented in Tables 2 and 3. The expression of each miRNA was measured and normalized to that of miR295 as a control. Results are expressed as the mean ± SD (*n* = 3).
(TIF)

Table S1 **Classification of annotated small RNAs in male and female blastocysts against RepeatMasker and tRNA database.**
(XLS)

Table S2 **A list of annotated small RNAs expressed in male and female blastocysts.**
(XLS)

Table S3 **A list of the PCR primer sets used in this research.**
(XLS)
