# Supplementary figures and images for: Identification of an Imprinted Gene Cluster in the X-Inactivation Center
Source: PLoS One. 2013 Aug 6;8(8):e71222. doi: 10.1371/journal.pone.0071222 (PMC3735490; doi:10.1371/journal.pone.0071222)

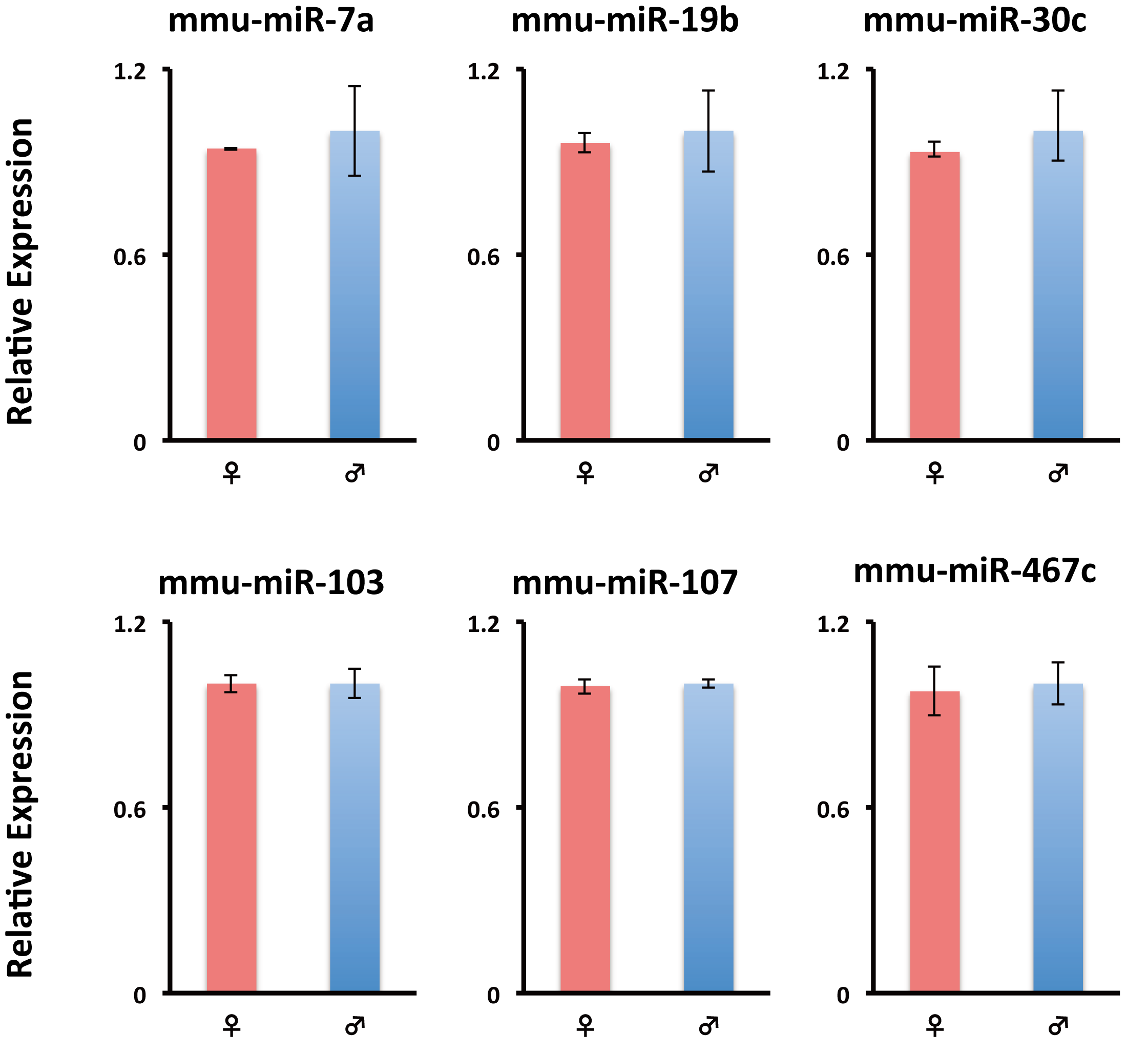

Supplement: Figure S1 — Validation of the expression of micro RNAs using qRT–PCR. The qRT–PCR analysis of differentially expressed microRNA candidates mmu-miR-7a, mmu-miR-19b, mmu-miR-30c, mmu-miR-103, mmu-miR-107 and mmu-miR-467 that are presented in Tables 2 and 3. The expression of each miRNA was measured and normalized to that of miR295 as a control. Results are expressed as the mean ± SD (n = 3). (TIF) [file pone.0071222.s001.tif]
